# Aphid Feeding Induces Phytohormonal Cross-Talk without Affecting Silicon Defense against Subsequent Chewing Herbivores

**DOI:** 10.3390/plants9081009

**Published:** 2020-08-10

**Authors:** Scott N. Johnson, Rhiannon C. Rowe, Casey R. Hall

**Affiliations:** Hawkesbury Institute for the Environment, Western Sydney University, Penrith NSW 2751, Australia; Rhiannon.Rowe@westernsydney.edu.au (R.C.R.); Casey.Hall@westernsydney.edu.au (C.R.H.)

**Keywords:** aphid, caterpillar, chewing, jasmonic acid, plant defense, salicylic acid, sap-sucking, silica, silicon

## Abstract

Prior feeding by insect herbivores frequently affects plant quality for herbivores that subsequently feed on the plant. Facilitation occurs when one herbivore improves plant quality for other herbivores, including when the former compromises plant defenses. Silicon (Si) is an important defense in grasses that increases following activation of the jasmonic acid (JA) pathway. Given that aphids often stimulate the salicylic acid (SA) pathway, we hypothesized that this could reduce Si defense because of the well documented antagonistic cross-talk between SA and JA. We tested this in the model grass *Brachypodium distachyon* with and without Si (+Si and −Si, respectively); half of the plants were exposed to aphids (*Rhopalosiphum padi*) and half remained aphid-free. Aphid-free and aphid-exposed plants were then fed to chewing herbivores (*Helicoverpa armigera*). Aphids triggered higher SA concentrations which suppressed JA concentrations but this did not affect foliar Si. Chewing herbivores triggered higher JA concentrations and induced Si uptake, regardless of previous feeding by aphids. Chewer growth rates were not impacted by prior aphid herbivory but were reduced by 75% when feeding on +Si plants. We concluded that aphids caused phytohormonal cross-talk but this was overridden by chewing herbivory that also induced Si uptake.

## 1. Introduction

Plant-mediated interactions between herbivorous insects are common in nature and are often driven by herbivore-induced changes in plant nutritional and defensive chemistry. The majority of these interactions are competitive but >11% represent facilitation whereby feeding by one herbivore increases the performance of herbivores that subsequently feed on the plant [1,2]. The mechanisms for facilitation may be complex but can involve the first herbivore compromising how plants subsequently deploy defenses, especially when the two herbivores are from different feeding guilds (e.g., phloem feeding and leaf chewing) [3,4]. This arises because of interference between defense signaling pathways in the plant [3] but see Reference [4].

Herbivory activates complex signaling pathways involving several phytohormones, including jasmonic acid (JA), salicylic acid (SA) and ethylene, which regulate expression of defense genes and downstream production of defensive compounds [5,6,7,8]. The JA pathway is viewed as the master regulator of plant resistance to arthropod herbivores and pathogens [8,9,10]. Broadly speaking, JA regulates defenses against tissue-chewing insect herbivores, whereas defenses against fluid-feeding insects (i.e., piercing/sucking) herbivores are regulated by both SA and JA pathways [11,12]. In addition to having separate biosynthetic origins, the SA and JA pathways utilize separate compounds for signal transduction [11]. In particular, activating the SA pathway often suppresses the activity of JA pathway and vice versa, via antagonistic cross-talk at several biosynthetic nodes [11,13]. This cross-talk fine-tunes which defensive responses are deployed against specific attackers, especially in terms of herbivore feeding guild [14]. This negative SA-JA crosstalk can drive facilitation with several reports of aphid-infestation promoting performance of caterpillars because SA signaling interferes JA-induced plant defenses [3,15,16].

Grasses have a comparatively small repertoire of chemical defenses against herbivores [17,18], which instead rely on accumulating large amounts of silicon (Si) from the soil which is then deposited in tissues [19,20]. There are a few examples of Si-mediated indirect defense (i.e., involving recruitment of the herbivores natural enemies) [21,22] but the majority of reports involve direct defenses [20]. The mechanisms of herbivore defense vary but Si deposition between and within cell walls most likely confers physical resistance to herbivory [23,24]. Moreover, Si may augment leaf trichomes/hairs and form discrete structures (e.g., opaline phytoliths) on the leaf surface [25,26]. These can interfere with feeding, wear down mouthparts and reduce nutrient acquisition by herbivores once ingested [27,28], although these effects vary between plant species [29]. Moreover, several studies report that Si is an inducible defense which increases in the plant following herbivore attack [30,31,32,33], demonstrably within 24 h of the JA pathway been activated [34].

While there are some key differences between studies [35], there is consistent evidence that Si defenses are positively linked to the JA pathway [34,35,36,37,38]. In particular, stimulation of the JA pathway increases Si defenses in both rice [37] and *Brachypodium distachyon* [34,35]. Given that sap-sucking insects (e.g., aphids) often trigger the SA pathway, potentially suppressing the JA pathway via cross-talk, this raises the prospect that aphid herbivory could compromise a plants ability to deploy Si defenses against subsequent herbivore attack. No studies, to our knowledge, have addressed Si-mediated facilitation between herbivores.

The objective of this study was to establish whether feeding by a phloem-feeding herbivore, the aphid (*Rhopalosiphum padi*), facilitates subsequent herbivory by the caterpillar (*Helicoverpa armigera*) by compromising Si defenses. We hypothesized that feeding by aphids stimulates SA and suppresses JA via cross-talk and this reduces Si concentrations in the leaves. As a consequence, JA responses and Si induction is lower in plants previously exposed to aphids which consequently ameliorates the negative impacts of Si on caterpillars.

## 2. Results

### 2.1. Experiment Summary

**Figure 1:**
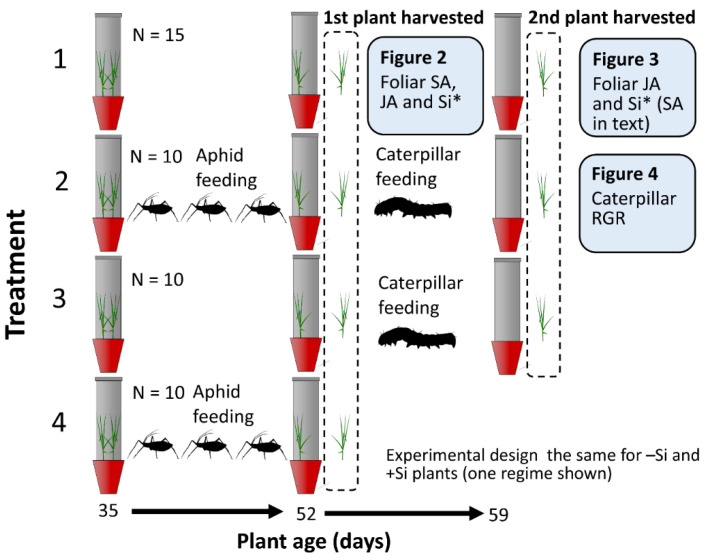
Schematic of the experimental design summarizing the sequence of treatments, replication and how these relate to results in subsequent figures. The full experiment was replicated for −Si and +Si plants (one regime shown for clarity). * Si concentrations analyzed on +Si plants only since −Si plants were not supplied with Si.

The study involved exposing plants to aphids to determine their impacts on phytohormones and Si concentrations relative to aphid-free plants (Section 2.2). We then presented these plants to caterpillars in no-choice feeding assays (Section 2.3). This is summarized in Figure 1 and described fully in Materials and Methods (Section 4).

### 2.2. Impacts of Aphid Feeding on Phytohormones and Si

**Figure 2:**
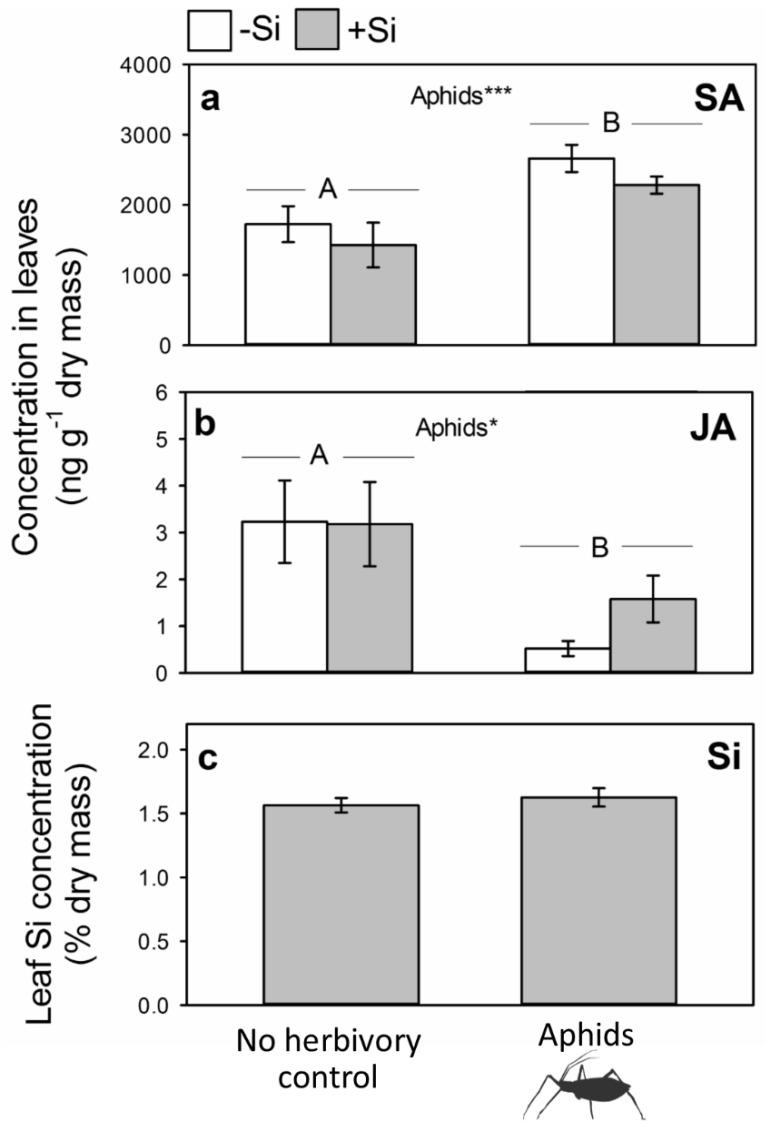
Leaf concentrations of (**a**) salicylic acid (SA), (**b**) jasmonic acid (JA) in −Si and +Si plants and (**c**) Si (+Si plants only) subjected to aphid herbivory prior to feeding by caterpillars. Results of statistical analysis (**a**) Si: F_1,28_ = 2.08, *p* = 0.160; Aphids: F_1,28_ = 14.61, *p* < 0.001; Si × Aphids: F_1,28_ = 0.03, *p* = 0.861; (**b**) Si: χ_1,28_ = 0.12, *p* = 0.726; Aphids: χ_1,28_ = 6.21, *p* = 0.013; Si × Aphids: χ_1,28_ = 0.03, *p* = 0.142 and (**c**) Aphids: F_1,28_ = 0.46, *p* = 0.504. Significant treatments indicated *** *p* < 0.001 and * *p* < 0.05 and uppercase letters used to indicate differences between treatments. Mean ± SE shown.

Initially we determined the effects of three bouts of aphid herbivory on phytohormone and Si concentrations relative to control (aphid-free) plants. Three bouts of herbivory were used to maximize the likelihood of us detecting aphid-induced changes in the plant. Plants were phenotypically similar, regardless of treatment, with plant biomass being unaffected by Si supply (F_1,47_ = 1.83, *p* = 0.182), aphid herbivory (F_1,47_ = 2.76, *p* = 0.103) or the interaction of the two treatments (F_1,47_ = 0.80, *p* = 0.375). Concentrations of SA were significantly higher in plants that had been exposed to aphids (Figure 2a), which corresponded to a significant decrease in JA (Figure 2b). There was a strong negative correlation between SA and JA concentrations (r_s_ = −0.471, *p* = 0.007), which further suggests that aphids were causing antagonistic cross-talk between the two hormones. Si supply had no effect on either SA or JA and there was no interaction between aphid herbivory and Si status. Analysis of Si concentrations was conducted on +Si plants only since Si levels were below detectable limits (< 0.3%) in −Si plants. Aphids had no impact on leaf Si concentrations (Figure 2c).

### 2.3. Subsequent Changes in JA and Si When Attacked by Caterpillars

**Figure 3:**
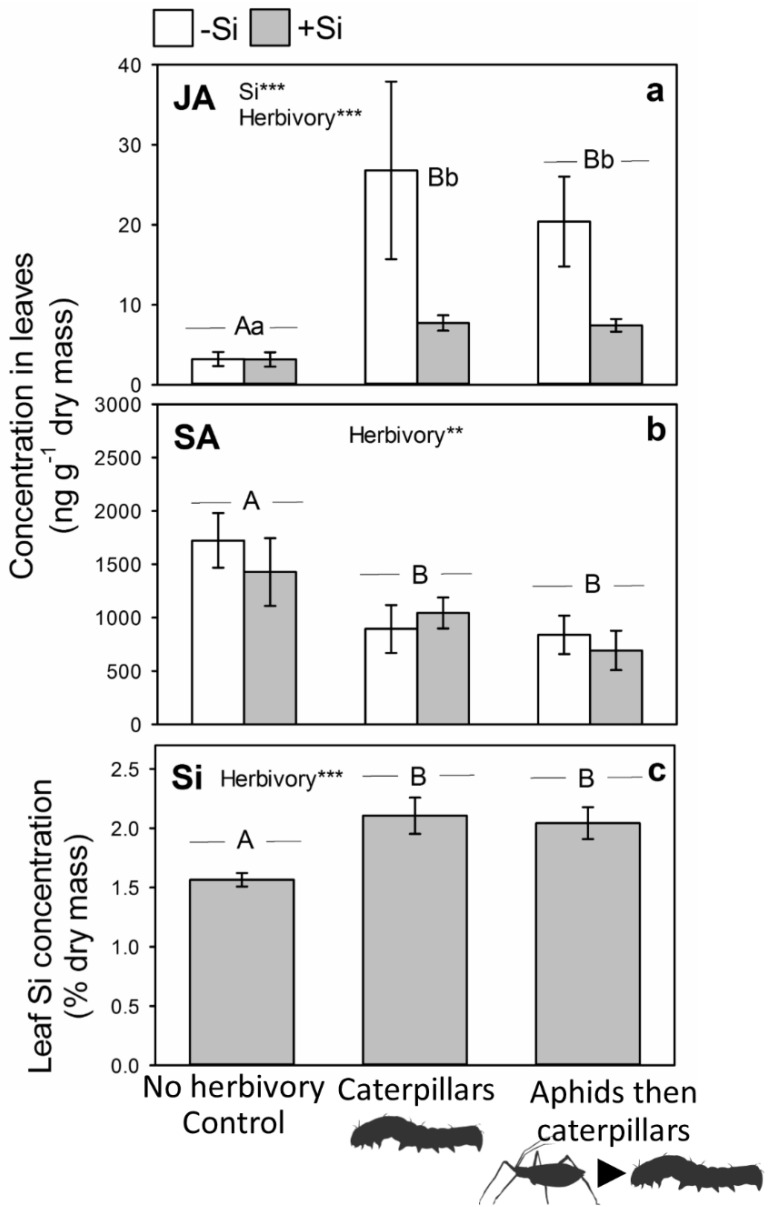
Impacts Si and previous aphid feeding on leaf (**a**) JA, (**b**) SA and (**c**) Si concentrations following herbivory by caterpillars. Results of statistical analysis: (**a**) Si: χ_1,42_ = 10.73, *p* = 0.001; Herbivory: χ_2,42_ = 16.99, *p* < 0.001; Si × Herbivory: χ_2,42_ = 0.32, *p* = 0.852; (**b**) Si: χ_1,42_ = 0.09, *p* = 0.606; Herbivory: χ_2,42_ = 4.45, *p* = 0.004; Si × Herbivory: χ_2,42_ = 0.30, *p* = 0.654; (**c**) Herbivory: F_2,32_ = 8.11, *p* = 0.001. Significant treatments indicated *** *p* < 0.001 and ** *p* < 0.01 and uppercase letters used to indicate differences between herbivore treatments and lowercase letters between Si treatments. Mean ± SE shown.

Having harvested plants to determine the impacts of aphid-feeding, we presented the remaining control (aphid-free) and aphid exposed plants to caterpillars in no-choice feeding assays for 7 days before these were harvested. We observed that caterpillars triggered higher JA concentrations relative to caterpillar-free plants but to a similar extent, regardless of whether plants had previously been exposed to aphids (Figure 3a). There was a significant effect of Si supply on JA concentrations but this was largely driven by the more substantive increase in JA in −Si plants challenged by the caterpillars (Figure 3a), which we have previously observed [34,35]. Caterpillar herbivory significantly reduced leaf SA concentrations, regardless of prior aphid feeding (Figure 3b), lending further support that phytohormal cross-talk was occurring. Si had no impact on SA concentrations and there was no interaction between Si supply and herbivory (Figure 3b). Like the JA response to caterpillars, we observed that Si uptake significantly increased relative to caterpillar-free plants but Si induction was not affected by previous exposure to aphids (Figure 3c).

### 2.4. Caterpillar Performance

**Figure 4:**
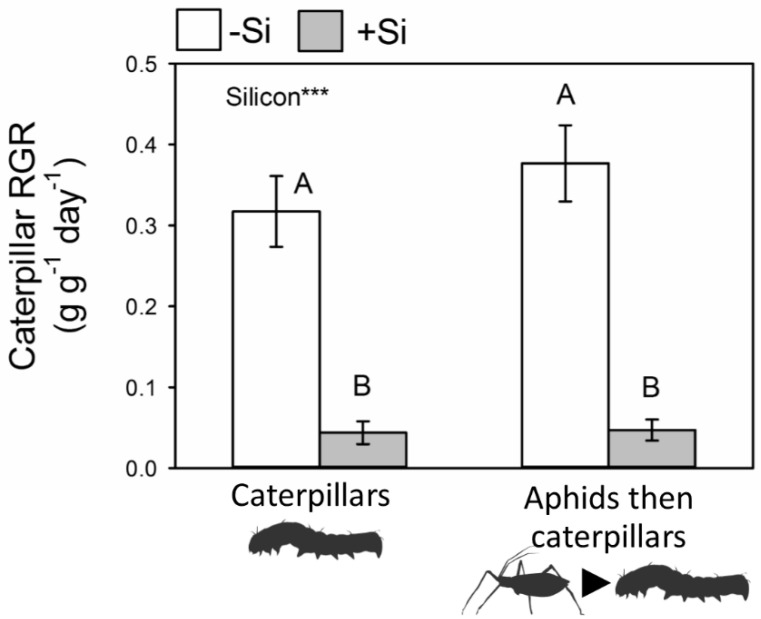
The effects of Si and previous aphid herbivory on relative growth rates (RGR) of *H. armigera*. Results of statistical analysis: Si: F_1,36_ = 59.54, *p* < 0.001; Aphids: F_1,36_ = 1.83, *p* = 0.185; Si × Aphids: F_1,36_ = 0.18, *p* = 0.675. Significant treatments indicated *** *p* < 0.001 and uppercase letters used to indicate differences between treatments. Mean ± SE shown (*n* = 10).

*Helicoverpa armigera* relative growth rates (RGR) decreased by 75% when feeding on +Si plants but previous feeding by aphids had no impact (Figure 4). There was no interactive effect of Si treatment and prior feeding by aphids.

## 3. Discussion

This study demonstrated that aphid herbivory changes concentrations of defense hormones in a manner that is consistent with antagonistic cross-talk. Despite aphids depressing JA concentrations, however, which we hypothesized would lower Si uptake, we observed that caterpillars continued to trigger higher JA concentrations and Si uptake regardless of previous aphid feeding. The JA response following caterpillar herbivory appears to override the previous suppression of JA by aphids and as a consequence aphids had no impact on the performance of caterpillars.

There are now numerous studies that have investigated how prior herbivory affects the performance of subsequent herbivores, especially using contrasting feeding guilds. The results are highly variable, however, with aphid-infestation having positive [3,15,16], neutral [39,40,41] or negative [42] impacts on caterpillars that subsequently feed on the plant. Even within the same study, results can be mixed. For example, aphids (*Brevicoryne brassicae*) positively affected the specialist caterpillar (*Plutella xylostella*) but not the generalist (*Mamestra brassicae*) [16]. Aphids (*Aphis neri*) positively affected monarch caterpillars (*Danaus plexippus*) on one species of milkweed (*Asclepias syriaca*) but not another (*A. tuberosa*) [15]. While aphids (*Myzus persicae*) temporarily facilitated the performance of a chewing herbivore (*Leptinotarsa decemlineata*) in the field, this was not replicated in laboratory assays [43]. Most of these studies were conducted in model dicot species, especially the Brassicaceae and to our knowledge none have studied sequential herbivory interactions in grasses. The grasses are generally much more reliant on Si for herbivore defense than these model species, so it would be particularly disadvantageous for Si defense to be deactivated. This may explain why induction of Si accumulation appears to be robust in *B. distachyon*, despite aphids initially suppressing JA concentrations.

While *R. padi* stimulated SA concentrations in *B. distachyon* in this study, this may not necessarily be related to a defensive response in the plant. In particular, triggering the SA pathway sometimes confers inconsistent defense against aphids and that this may even be a mechanism that aphids deploy to counteract more potent JA defenses [12]. In other words, some aphids may be manipulating plant defenses for their own benefit using the plant’s own hormonal cross-talk [12]. Moreover, previous studies have reported diminished aphid performance when feeding on milkweed and tomato plants because of chewer-induced levels of JA defenses [15,44]. In summary, stimulation of the SA pathway may not have caused significant changes in the plant, whereas activation of the JA pathway (even at low levels) following caterpillar herbivory was more effective, particularly in terms of Si accumulation. In support of this, the exogenous application of methyl jasmonate (which stimulates the JA pathway) to Si supplemented *B. distachyon* plants resulted in lower JA increases than Si-free plants but still increased foliar Si concentrations in soil [35] and hydroponic conditions [34]. Si accumulation typically resulted in changes in leaf surface morphology, such as leaf macro-hairs [35], silica cells and prickle cells [26] which have been linked to reduced caterpillar performance [26]. We did not quantify Si concentrations in the roots, so it is not clear if Si induction arose from increased Si uptake, remobilization of Si from the roots to the leaves or both. However, given the large increase in leaf Si following caterpillar damage (30–35%) it seems likely that increased Si uptake was involved.

Methodologically, we applied three bouts of aphid herbivory to maximize the likelihood that they would cause phytohormonal changes. From daily inspection, we did not observe any difference in aphid feeding activity between first and third bouts of herbivory, which suggests aphids were not being adversely affected by induced defenses. This is consistent with our previous observations that Si defenses in +Si *B. distachyon* are ineffective against *R. maidis* [45], which has also been reported for other aphid species feeding on other grasses [27,46]. This was also our rationale for hypothesizing that aphids have more of impact on chewing herbivores via silicon defenses than vice versa.

The aphid clone, feeding intensity and plant accession used in the current study caused the predicted increase and decrease in SA and JA concentrations, respectively. It should be noted that in other systems, different aphid clones affect these phytohormones to differing extents [47], so this may not occur for other *R. padi* clones. This does not affect our conclusions, however, since Si defenses operated regardless of the SA-JA crosstalk so it seems reasonable to assume this would have been the case in the absence of this crosstalk.

As previously reported [34,35], we observed that the increase in JA following chewing herbivory was higher in −Si *B. distachyon* plants than +Si plants. We interpreted this is because Si enrichment means plants have constitutive physical defenses in place and therefore have less utility for secondary metabolite based defenses [35]. This is consistent with several studies that have reported a negative correlation between silicon defenses and carbon-based defenses, such as phenolics and tannins [48,49,50,51]. Nonetheless, we still have a limited understanding of how silicon defenses against herbivores relate to defensive phytohormonal signaling, particularly in terms of gene expression. We acknowledge that our study is somewhat exploratory in this regard.

The use of Si for herbivore defense has been widely advocated for above- [19] and belowground [52] herbivores. This is germane given that just three grass species directly supply the world’s population with 42% of their calorie intake [53], all of which take up significant amounts of Si [54]. The current study suggests that Si defenses are durable and persist even if previous feeding by aphids depresses JA via phytohormonal cross-talk.

## 4. Materials and Methods

### 4.1. Plants and Herbivores

*Brachypodium distachyon* (Bd21-3), supplied by the French National Institute for Agricultural Research (INRA, Versailles, France), were grown hydroponically using the system and procedures outlined by Hall et al. [34]. Two plants were grown in opaque cups filled with 330 mL nutrient solutions. Silicon inclusion for +Si plants was achieved by adding liquid potassium silicate (K_2_SiO_3_) (Agsil32, PQ Australia, SA, Australia) at a concentration of 2 mM (SiO_2_ equivalent) to the nutrient solution and adjusted to pH 5.5 using HCl to reduce the polymerization of silicates [55]. Silicon-free (−Si) plants had KCl added to balance additional K^+^ and Cl^−^ in the Si^+^ treatments and adjusted to pH 5.5 using HCl. Cups were also rotated within the glasshouse chamber and solutions replaced weekly. Plant propagation and experiments were conducted in naturally lit glasshouse chambers maintained at 22/18 °C Light:Dark on a 14:10 h cycle. Humidity was controlled at 50% (± 6%). Conditions were monitored and regulated using the PlantVisorPRO (Carel Industries, Padova, Italy) system.

Aphid (*Rhopalosiphum padi* L.) cultures were established from a single parthenogenetic female obtained from laboratory cultures at Agriculture Victoria Research (Horsham, VIC, Australia) and reared on barley plants (*Hordeum vulgare* cultivar ‘Hindmarsh’). The caterpillar (*Helicoverpa armigera* Hübner), supplied by CSIRO Agriculture & Food, Narrabri Australia, were fed on growing media (modified from Teakle & Jensen [56]) at 20 °C 15:9 h photoperiod (Light:Dark) until required.

### 4.2. Experimental Procedure

Ninety cups, each containing two plants, were grown hydroponically for five weeks, half in −Si solution and half in +Si solution. Plants were assigned at random to four treatments (Figure 1) comprising: (treatment 1) no herbivory (control) (*n* = 15), (treatment 2) three bouts of aphid herbivory, then caterpillars (aphids then caterpillars) (*n* = 10), (treatment 3) no aphids, then caterpillars (caterpillars) (*n* = 10) and (treatment 4) three bouts of aphid herbivory (aphids) (*n* = 10). Three bouts of aphid herbivory were applied to maximize the likelihood of induced changes in the plants. Repeated bouts of aphid herbivory began when plants were 35 days old, by applying either four adult aphids for three days, removed for four days, then re-applied using new insects. Cups were checked daily to ensure that both plants were experiencing aphid herbivory.

When plants were 52 days old, one plant from each cup was harvested by snap-freezing tissue in liquid nitrogen and stored at −80 °C prior to chemical analysis (see below). All plants were weighed following freeze-drying. The remaining plants were grown for a further week with individual third instar *H. armigera* applied to 10 −Si and 10 +Si cups that had not been exposed to aphids (treatment 3; Figure 1) and plants with previous aphid herbivory (treatment 2; Figure 4). Third instar larvae that had just emerged from second instar were used since, while relatively small, they can be easily transferred to plants without mortality [33]. Relative growth rates (RGR) were calculated [mass gained (mg)/initial mass (mg)/time (days)] by measuring larval mass upon application and removal from the plant 7 d later. Throughout the experiment, all plants (including herbivore-free plants) were caged using transparent cylinders with mesh apertures (similar to those described in Johnson et al. [33]) fixed to the cups.

### 4.3. Si Analysis

Approximately 80 mg of ground leaf material from all plants was analyzed to measure Si concentration using an X-ray fluorescence spectrometer (Epsilon 3x; PANalytical, EA Almelo, The Netherlands) following the procedure of Reidinger et al. [57]. Analysis was calibrated using plant material (NCS ZC73018 Citrus leaves, China National Institute for Iron and Steel) of known Si concentrations (see Hiltpold et al. [58] for full details).

### 4.4. JA and SA Analysis

Deuterated JA (d5-JA) and SA (d4-SA) internal standards were purchased from CDN Isotopes (Quebec, Canada) and OlChemIm Ltd. (Olomouc, Czech Republic), respectively. HPLC grade methanol, chloroform and phytohormone calibration standards were purchased from Sigma-Aldrich (St. Louis, MO, USA).

Frozen material from eight replicates of every treatment were selected at random and freeze dried for further analysis. The phytohormones salicyclic acid (SA) and jasmonic acid (JA) were extracted using the Bligh-Dyer method [59] to remove interfering compounds from the plant matrix. In brief, 50 mg of ground leaf material was extracted with 500 uL of 70% methanol spiked with d5-JA and d4-SA as internal standard to yield a final concentration of 100 ppb. Samples were mixed for 30 min at 4 °C in a rotator mixer, 180 uL of chloroform was added and samples vortexed for 30 s. This was repeated with another 180 uL of chloroform and then 200 uL of water was added, samples were then centrifuged at 6000 rpm for 10 min at room temperature, resulting in a triphasic system. The upper water/methanol layer was carefully transferred to a clean 2 mL Eppendorf tube and passed through a 0.22 µm Polytetrafluoroethylene (PTFE) filter. The extracts were analyzed by ultrahigh performance liquid chromatography-electrospray ionization tandem mass spectrometry (UPLC/ESI-MS/MS) using an Acquity UHPLC coupled to a Xevo triple quadrupole mass spectrometer (Waters Corporation, Milford, MA, USA). Five microliters of extract were injected into a 2.1 mm × 50 mm × 1.7 µm, C18 reverse phase column. The mobile phase was composed of water (A) and acetonitrile (B) both containing 0.1% (*v/v*) formic acid at a constant flow rate of 0.6 mL min^−1^. Elution was performed as a linear gradient (A%, t min): 80% A at 0 min; 50% A at 2 min; 0% A at 2.1 min. Phytohormones were detected by ESI-MS/MS operating in negative ion mode. JA and SA identification was based on the fragmentation pattern as compared with authentic standards. Quantification was based on a calibration curve of the standards and adjusted for sample recovery based on the internal standards. Final concentrations were standardized by dry weight of the sample.

### 4.5. Statistical Analysis

Leaf SA concentrations from the first harvest (Figure 1) conformed to normality and were analyzed with a two-way ANOVA with Si treatment and aphid presence/absence and their interaction, included as fixed factors. Leaf SA concentrations from the second harvest (Figure 1) were analyzed with a generalized linear model with a gamma distribution and reciprocal link function. JA concentrations were analyzed with a generalized linear model with negative binomial distribution and log-ratio link function, due to over dispersion, using the same factors as above. Potential relationships between SA and JA were examined using a Spearman’s rank correlation test. Si concentrations (in +Si plants only) conformed to normality and were analyzed with a one-way ANOVA with aphid presence/absence included as the fixed factor. *Helicoverpa armigera* RGR was analyzed with a two-way ANOVA with Si treatment and prior aphid herbivory and their interaction, included as fixed factors. Where a factor was statistically significant, differences between treatments were identified using Tukey’s post hoc tests. All analysis was conducted in Genstat (v19), Hemel Hempstead, UK.
